# Reducing FKBP51 Expression in the Ventral Hippocampus Decreases Auditory Fear Conditioning in Male Rats

**DOI:** 10.3390/ijms25137097

**Published:** 2024-06-28

**Authors:** Nashaly Irizarry-Méndez, Marangelie Criado-Marrero, Anixa Hernandez, Maria Colón, James T. Porter

**Affiliations:** 1 Department of Basic Sciences, Ponce Research Institute, Ponce Health Sciences University, Ponce 00716, Puerto Rico; nirizarry20@stu.psm.edu (N.I.-M.);; 2 Department of Neuroscience, University of Florida, Gainesville, FL 32610, USA; mcriadomarrero@ufl.edu

**Keywords:** ventral hippocampus, FKBP5, glucocorticoids, stress, fear conditioning, fear extinction

## Abstract

Fear conditioning evokes a physiologic release of glucocorticoids that assists learning. As a cochaperone in the glucocorticoid receptor complex, FKBP51 modulates stress-induced glucocorticoid signaling and may influence conditioned fear responses. This study combines molecular and behavioral approaches to examine whether locally reducing FKBP51 expression in the ventral hippocampus is sufficient to affect fear-related behaviors. We hypothesized that reducing FKBP51 expression in the VH would increase glucocorticoid signaling to alter auditory fear conditioning. Adult male rats were injected with an adeno-associated virus (AAV) vector expressing short hairpin – RNAs (shRNA) targeting FKBP5 into the ventral hippocampus to reduce FKBP5 levels or a control AAV. Infusion of FKBP5-shRNA into the ventral hippocampus decreased auditory fear acquisition and recall. Although animals injected with FKBP5-shRNA showed less freezing during extinction recall, the difference was due to a reduced fear recall rather than improved extinction. Reducing ventral hippocampus FKBP51 did not affect exploratory behavior in either the open field test or the elevated zero maze test but did increase passive behavior in the forced swim test, suggesting that the reduction in auditory fear recall was not due to more active responses to acute stress. Furthermore, lower ventral hippocampus FKBP51 levels did not alter corticosterone release in response to restraint stress, suggesting that the reduced fear recall was not due to lower corticosterone release. Our findings suggest FKBP51 in the ventral hippocampus plays a selective role in modulating fear-learning processes and passive behavioral responses to acute stress rather than hypothalamic-pituitary-adrenal axis reactivity or exploratory responses.

## 1. Introduction

The FK506-binding protein 51 (FKBP51), encoded by the FKBP5 gene, is an Hsp90 co-chaperone involved in glucocorticoid receptor (GR) signaling [1]. In the rat brain, GRs are enriched in the hippocampus [2]. The hippocampus, a critical component of the limbic system, plays a vital role in forming and retrieving memories, which are intricately linked to emotions and behavioral responses [3,4]. Studies have shown that hippocampal alterations could lead to neuropsychiatric disorders in which memory is compromised [5]. 

Glucocorticoids play a central role in adaptive responses to stressors [4,6,7]. Aversive learning induces an increase in glucocorticoids in the brain, which bind to intracellular GRs. The active GR complex enters the nucleus to alter gene transcription and enhance memory formation [1]. In the hippocampus, this process involves a complex signaling pathway. GR activation by glucocorticoids stimulates the expression of the immature form of brain-derived neurotrophic factor (pro-BDNF) and tissue plasminogen activator (tPA). The increased tPA cleaves pro-BDNF into its mature form (m-BDNF). m-BDNF then activates the tyrosine kinase receptor B, leading to the phosphorylation of ERK1/2 MAPK intracellular kinases. This phosphorylation increases the expression of the downstream transcription factors and proteins that enhance memory encoding within the hippocampus [8].

FKBP5 modulation is another mechanism by which GR signaling is hypothesized to control memory [9]. GR signaling enhances the transcription of FKBP5, producing a negative feedback loop in which increased FKBP51 expression inhibits GR translocation to the nucleus, thereby terminating GR-mediated transcription and reducing the sensitivity of cells to glucocorticoids [10]. Single nucleotide polymorphisms in the FKBP5 gene have been associated with the development of fear-related disorders [11,12,13,14], suggesting that dysregulation of FKBP51 expression could alter fear learning and memory. FKBP51 expression might also regulate fear memory via GR-independent mechanisms, such as modulation of the phophoinositide 3-kinase (PI3K)/protein kinase B (PKB or AKT)/mammalian target of rapamycin (mTOR) pathway [15,16,17]. However, few studies have examined the effects of altered FKBP51 expression on fear conditioning or extinction [18,19,20,21]. 

Since the ventral hippocampus (VH) is particularly sensitive to the effects of stress [22,23,24,25] and modulates fear learning [26,27,28], FKBP51 expression could alter the hippocampal function to affect fear-related behavior. Stress-evoked glucocorticoid signaling can directly target and induce changes in hippocampal neurons to regulate memory consolidation [1,29,30,31]. Moderate levels of glucocorticoids (like cortisol or corticosterone) enhance memory consolidation [32,33,34], while excessive levels of glucocorticoids impair memory [35,36,37,38]. At the cellular level, low concentrations of corticosterone increase the excitability of hippocampal neurons, while high concentrations depress hippocampal activity [23,39,40]. Lower levels of FKBP51 could enhance glucocorticoid signaling to strengthen conditioned fear memory through the modulation of NMDA and AMPA receptors [22,24,41,42,43]. However, excessive glucocorticoid-induced plasticity could weaken fear memory [44]. Therefore, we hypothesized that sustained low levels of FKBP51 protein in the hippocampus will lead to a weakening of conditioned fear memory due to loss of specific learning-related plasticity. 

In this study, we examined whether reducing FKBP51 expression in the VH is sufficient to affect fear-related behaviors. Viral knockdown of FKBP51 in the VH reduces auditory fear learning and increased passive coping behaviors. In contrast, locomotion, exploratory behavior, and the reactivity of the HPA axis to acute restraint stress were unaffected by reducing VH FKBP51. 

## 2. Results

### 2.1. Decreasing FKBP51 Expression in the VH Reduces Fear Acquisition and Recall

**Figure 1:**
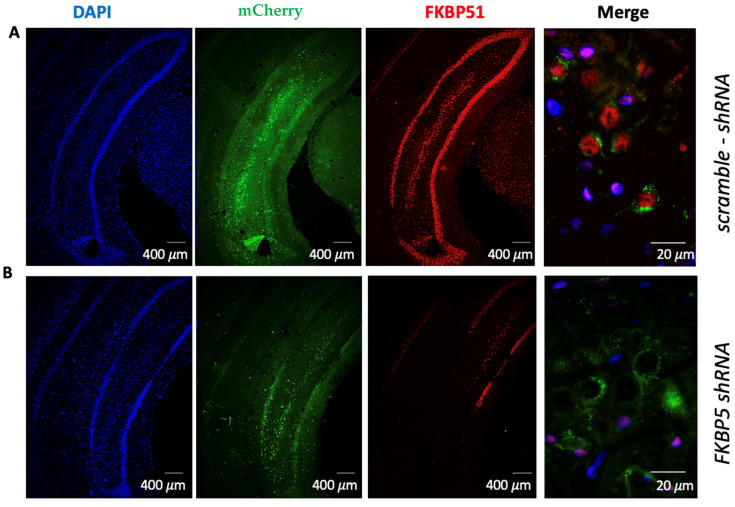
Infusion of AAV5 expressing FKBP5 shRNA reduced expression of FKBP51 protein in the VH. (**A**) Fluorescent images show that localized expression of mCherry in the VH one month after infusion of AAV5 expressing scramble-shRNA did not alter FKBP51 expression. The higher magnification image to the right shows expression of FKPB51 in cells expressing mCherry. (**B**) Localized expression of mCherry in the VH one month after infusion of AAV5 expressing FKBP5 shRNA shows lack of FKBP51 expression in areas expressing mCherry. The higher magnification image to the right shows a lack of FKBP51 in cells expressing mCherry. Dapi (blue), mCherry (green), FKBP51 (red).

In the first set of experiments, we evaluated whether reducing the FKBP51 protein in the VH is sufficient to alter fear conditioning or extinction. First, we verified that shRNA against FKBP5 would decrease the expression of FKBP51 in the VH. Adult male rats were injected with either a control AAV5 expressing a scramble shRNA or an AAV vector expressing four distinct shRNAs targeting FKBP5 and the fluorescent marker mCherry into the VH. By using an AAV5, we ensured efficient transduction in hippocampal neurons and glial cells [45]. One month later, the infusion site and viral efficiency were verified with immunofluorescence labeling of mCherry and FKBP51 (Figure 1). Pyramidal cells in layers CA1 and CA3 showed prominent expression of mCherry. VH cells expressing the FKBP5 shRNA showed less expression of FKPB51, indicating that the shRNA effectively reduced the protein.

**Figure 2:**
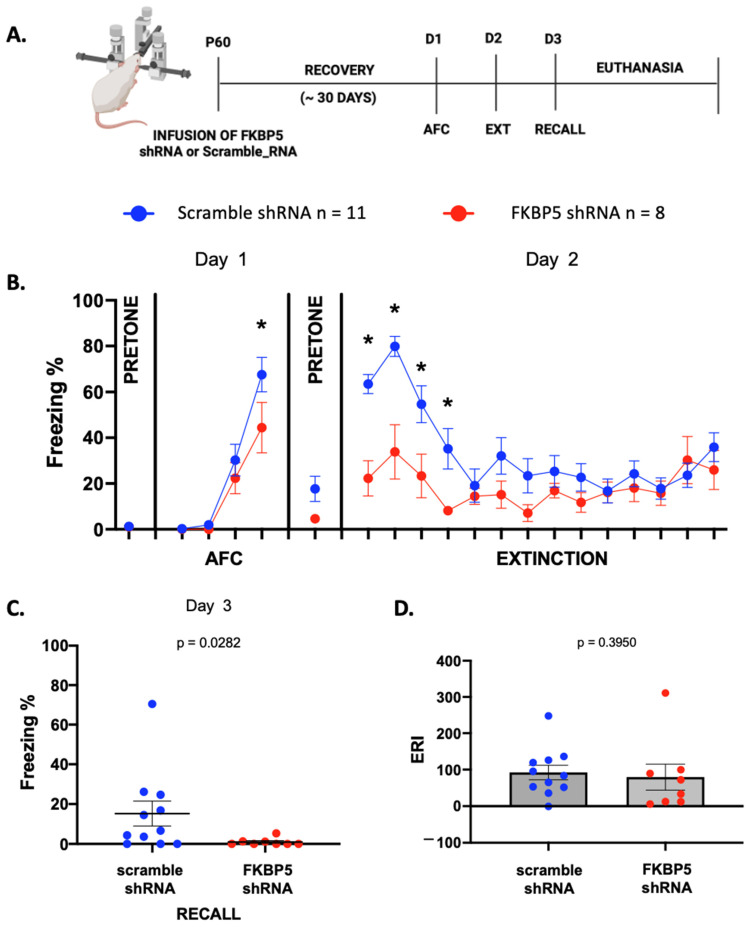
Reducing FKBP51 in the VH before auditory fear conditioning (AFC) reduces fear learning. (**A**) Timeline for behavioral training after VH infusions of AAV5-FKBP5-shRNA or AAV5-scramble-shRNA one month before AFC. (**B**) Percent of time rats spent freezing during AFC and extinction. Two-way ANOVA with repeated measures followed by a Fisher LSD test (*) *p* < 0.05. (**C**) Percent freezing of rats during extinction recall. (**D**) Reducing FKBP51 in the VH does not affect ERI. Mann–Whitney U test.

Subsequently, we examined whether reducing VH FKBP51 expression alters fear acquisition or extinction by exposing the male rats to auditory fear conditioning and extinction thirty days after infusing AAV expressing FKBP5-shRNA or a scramble-shRNA into the VH (Figure 2A). Only rats with confirmed infusion sites were included in the behavioral analysis. As shown in Figure 2B, rats infused with FKBP5-shRNA into the VH showed less freezing during fear conditioning and extinction. A repeated-measure two-way ANOVA indicates a main effect of treatment (F (1, 17) = 17.21, *p* = 0.0007) and tone (F (19, 323) = 13.79, *p* < 0.001) and a significant interaction between treatment and tone (F (19, 323) = 2.741, *p* = 0.0002). Infusion of the FKBP5-shRNA did not affect freezing to the context before the first tone (*p* = 0.9885) or to the first conditioning tone (*p* = 0.9784), indicating that reducing VH FKBP51 did not affect basal freezing to the context or the tone. However, decreasing VH FKBP51 before fear conditioning reduced the acquisition of the fear, leading to less freezing to the final conditioning tone (*p* = 0.0102). The following day, both groups showed similar amounts of contextual fear during the pretone period (*p* = 0.1454). FKBP5-shRNA animals froze less to the first four tones at the beginning of the extinction training, EXT 1 (*p* < 0.0001), EXT 2 (*p* < 0.0001), EXT 3 (*p* = 0.0005 and EXT 4 (*p* = 0.0027), indicating that reducing VH FKBP51 impaired recall of the auditory fear memory (Table 1). 

### 2.2. Effects of Decreasing VH FKBP51 Expression on Fear Extintion

At the end of the extinction training on day 2, the freezing levels were similar in both groups (*p* = 0.2644), suggesting that both groups learned extinction. On day 3, the group infused with FKBP5-shRNA showed a significant decrease in fear expression compared with the animals infused with the scramble-shRNA (Mann–Whitney test, U = 18.50, *p* = 0.0282), suggesting that reducing VH FKBP51 might enhance recall of extinction memory (Figure 2C). 

However, this difference in freezing during extinction recall could be due to a deficit in fear memory instead of an enhanced extinction recall. To test this, we calculated the extinction retention index (ERI), which has been used in human [46,47,48] and rodent studies [49,50]. This index expresses the extinction recall responses as a percentage of the fear responses acquired during the conditioning phase [51]. As depicted in Figure 2D, rats infused with FKBP5-shRNA showed a similar ERI compared to scramble-shRNA animals (Mann–Whitney test, U = 33, *p* = 0.3950). While the initial observation of decreased fear expression on extinction recall might imply an enhanced recall of extinction memory, the lack of significant difference in the ERI indicates that the reduction in freezing behavior could be attributed to factors other than an enhanced extinction recall.

### 2.3. Reducing FKBP51 in VH Does Not Affect Exploritory Behavior or Locomotion but Induces Passive Behavior during the Forced Swim Test

**Figure 3:**
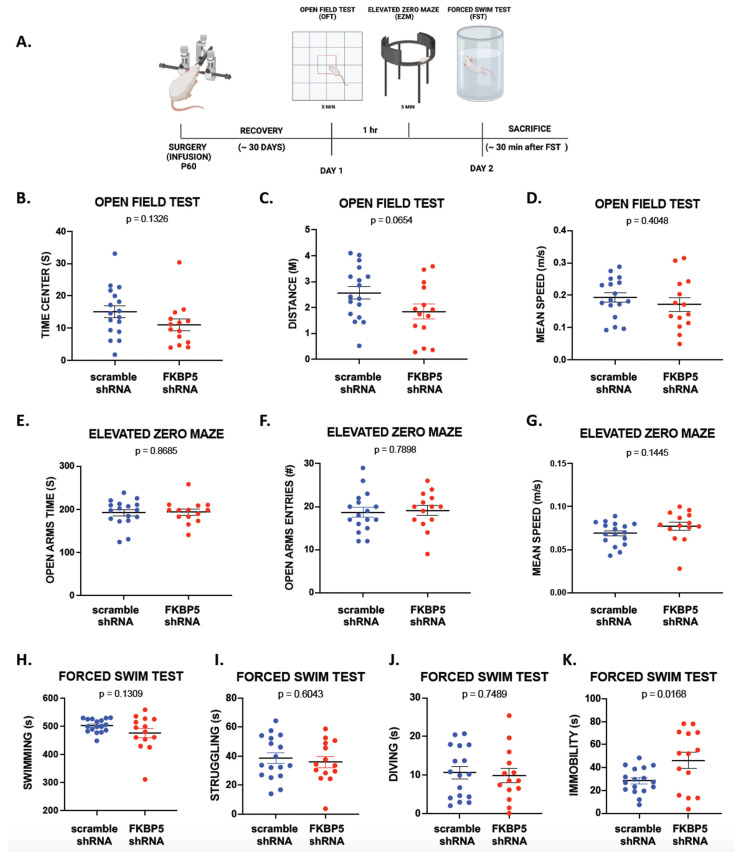
Reducing FKBP51 in the VH does not affect exploratory behavior or locomotion but increases passive behavior during the forced swim test. (**A**) Timeline for behavioral training one month after VH infusions of FKBP5 shRNA (n = 14) or scramble shRNA (n = 17). (**B**–**G**) Reducing FKBP51 in the VH has no effect on exploratory behavior during the OFT or EZM. (**H**–**K**) Reducing FKBP51 in the VH induces passive behavior during the forced swim test, reflected in increased immobility. Unpaired *t*-tests.

To examine the possibility that reducing FKBP51 in the VH decreases freezing behavior during auditory fear conditioning by increasing locomotion or exploratory behavior, we exposed a separate cohort of animals to an open field test (OF) and an elevated zero maze (EZM; Figure 3A). Figure 3B–D shows that reducing FKBP51 did not affect locomotion or exploratory behaviors, measured as distance traveled (t (29) = 1.915, *p* = 0.0654), mean speed (t (29) = 0.8455, *p* = 0.4048), or time spent in the center (t (29) = 1.547, *p* = 0.0740). Similarly, there was no effect on avoidant behaviors during the EZM test (Figure 3E–G). Exploratory behaviors were not affected, as measured by the number of entries to the open quadrants (t (29) = 0.2691, *p* = 0.7898), mean speed (t (29) = 1.500, *p* = 0.1445), and the time spent in the open quadrant (t (29) = 0.1670, *p* = 0.8685; Table 2). Our data suggests that FKBP51 in the VH is not a critical modulator of exploratory responses in these behavioral paradigms. 

Since mice lacking FKBP51 show more passive responses to the forced swim test (FST) [52], we examined whether exposing the FKBP5-shRNA rats to the acute stress of the FST would induce more passive behavior compared to the scramble-shRNA group (Figure 3A). As shown in Figure 3H–J, both groups showed similar swimming times (t (29) = 1.554, *p* = 0.1309), struggling times (t (29) = 0.5239, *p* = 0.6043), and diving times (t (29) = 0.3232, *p* = 0.7489). However, Figure 3K shows that animals infused with FKBP5-shRNA spent more time immobile (t (29) = 2.537, *p* = 0.0168). This suggests that VH FKBP51 may influence passive behavioral responses under stressful conditions, highlighting its potential involvement in the modulation of coping mechanisms.

### 2.4. Reducing FKBP51 in the VH Does Not Disrupt the HPA Axis Response to Acute Restraint Stress

**Figure 4:**
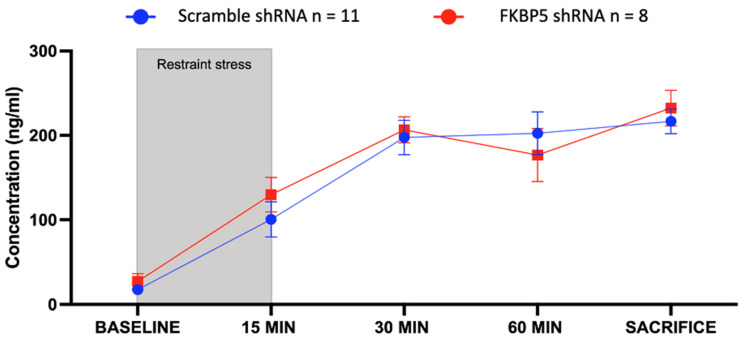
Reducing FKBP51 in the VH does not affect corticosterone response to restraint stress. Corticosterone levels in tail blood collected during and after restraint stress in rats infused with FKBP5-shRNA (n = 6) or scramble-shRNA (n = 6). Sacrifice blood was collected by cardiac puncture approximately 80 min after baseline. Two-way ANOVA with repeated measures. No difference was found between treatments (F (1, 10) = 0.1913, *p* = 0.6711).

To examine the possibility that changes in corticosterone levels disrupt the consolidation of fear memory, an additional cohort of animals were exposed to an acute stressor (15 min of restraint stress) after the FST. We collected blood from the tail to measure corticosterone in the serum of male rats expressing the scramble-shRNA or the FKBP5-shRNA in the VH at multiple time points during and after restraint stress (Figure 4). An additional sample was collected from the heart at sacrifice (approximately 80 min after restraint stress). Statistical analysis using a two-way ANOVA indicated no effect of treatment (F (1, 10) = 0.1913, *p* = 0.6711), suggesting that the reactivity of the HPA axis to acute stress is not altered by reducing FKBP51 in the VH.

## 3. Discussion

In this study, we sought to determine whether VH FKBP51 expression modulates the acquisition or extinction of auditory conditioned fear. We first introduce an AAV5 expressing shRNAs targeting FKBP5 mRNA into the VH of adult male rats to effectively decrease FKBP51 protein levels. Rats infused with FKBP5-shRNA exhibited reduced freezing responses during fear conditioning, fear recall, and extinction recall, indicative of impaired fear learning and memory. However, the reduction in FKBP51 in the VH did not alter baseline freezing to the first tone, suggesting that fear of novel stimuli was not altered. Similarly, exploratory behaviors remained unaffected by the reduction in VH FKBP51. Rats with decreased VH FKBP51 displayed increased immobility in the forced swim test, suggesting that FKBP51 might modulate passive behaviors to an acute stressor potentially through a distinct mechanism from fear memory acquisition and consolidation. Corticosterone serum levels were comparable between groups, indicating that reducing VH FKBP51 did not alter the reactivity of the HPA axis during acute restraint stress. Together, these findings suggest FKBP51 levels in the VH selectively modulate auditory fear learning and passive responses to acute stress rather than exploratory responses or HPA reactivity.

Since patients with PTSD have lower FKBP5 expression levels compared to trauma-exposed patients without PTSD [53,54], we used an animal model to mimic individuals who may have low FKBP51 levels in the VH prior to trauma. We found that manipulation of FKBP51 in the VH reduced fear responses during acquisition and impaired fear memory. Since VH activity is necessary for fear memory consolidation [26], reducing FKBP51 may have reduced the VH activity causing these behavioral responses. In support of this possibility, mice lacking FKBP51 expression show increased synaptic GABA release and reduced synaptic glutamate release into CA1 hippocampal neurons, which reduces hippocampal activity and hampers synaptic plasticity [52]. Mineralocorticoid receptors dampen glucocorticoid signaling by maintaining basal FKBP51 expression [55]. Without this regulation, excessive GR signaling could impair VH synaptic plasticity by increasing GABAergic inhibition [56]. Regardless of the mechanism, excessive GR signaling in the VH tends to impair auditory fear memory, since the infusion of corticosterone or dexamethasone into the VH reduced auditory fear conditioning [57].

The downregulation of FKBP51 protein in the VH of adult male rats weakened conditioned fear memory, suggesting that FKBP51 modulates the synaptic plasticity necessary for fear memory consolidation. Another explanation for the observed reduction in freezing responses could be attributed to increased glutamatergic activity, which creates synaptic noise, causing less synaptic specificity and a weaker associative memory [58]. By modulating GR signaling, FKBP51 levels may maintain the appropriate balance of synaptic excitation and inhibition required for the precise encoding of fear memories [59]. We also observed less freezing during extinction recall after reducing FKBP51 in the VH. The similar ERI between groups suggests that the observed decrease in freezing behavior in FKBP5-shRNA rats during extinction recall is likely due to a general reduction in fear response rather than an improved ability to recall extinction memory. This indicates that VH FKBP51 may play a crucial role in the acquisition and expression of fear memories, but its reduction does not necessarily facilitate the recall of extinction memory. Since the reconsolidation of fear memory is dependent on GR signaling [60], the decreased freezing during extinction recall in the FKBP5-shRNA animals could be mediated by impaired fear memory reconsolidation. However, further experiments are needed to test this hypothesis. 

The differences seen in auditory fear conditioning are not due to increased locomotion, or exploratory behaviors, since both groups showed similar behaviors in the open field test and elevated zero maze. Several studies have found that optogenetic manipulations and lesions of the VH affect exploratory behavior [61,62,63,64] and avoidance behaviors [65]. Conversely to these studies, we did not observe an effect of reduced FKBP51 in VH on anxiety-like behaviors or avoidance behaviors. These findings also suggest that the neural mechanisms within the VH associated with anxious behaviors or avoidance behavior may not be directly modulated by FKBP51. 

In addition to locomotion and exploratory tests, we used the FST to examine whether VH FKBP51 modulates active or passive responses to acute stress. We observed an increase in immobility in animals infused bilaterally with FKBP5-shRNA into the VH, suggesting a more passive behavior under acute stress. Our findings align with those of a study by Qiu and colleagues, who reported similar increases in immobility in mice lacking FKBP5 when subjected to the FST [52]. However, another study found no effect of depleting FKBP5 on basal coping behavior but increased active coping after restraint stress [66]. The absence of differences in swimming, struggling, or diving implies that differences observed in auditory fear conditioning after reducing FKBP51 in the VH were not due to a switch to more active responses to acute stress. 

Hippocampal projections innervate the paraventricular nucleus of the hypothalamus to modulate HPA reactivity to stress [67]. Given that glucocorticoids can modulate fear memory [68], we examined whether FKBP51 knockdown in the VH would result in differential serum corticosterone levels following acute stress exposure. Previous research by Touma and colleagues showed that global FKBP5 knockout dampens corticosterone responses to restraint stress [66]. However, we found that a localized knockdown of FKBP51 in the VH did not affect peripheral corticosterone responses. Aligned with our study, systemic pharmacological inhibition of FKBP51 with Safit2 did not alter plasma corticosterone levels after the FST [69]. Several factors may contribute to the discrepancy between our study and the findings reported by Touma and colleagues [66]. First, it is important to note that our study focused on a localized knockdown of FKBP51 within the VH, while theirs investigated global knockout mice, which may have broader effects on HPA axis regulation. Another potential explanation of our findings could be related to the timing of our assessments. We chose to sacrifice the animals 20 min after the last time point of blood collection, which is insufficient to observe a return to baseline corticosterone levels. It is possible that if we had extended the observation period, we might have observed a delayed or prolonged effect on corticosterone levels associated with FKBP51 knockdown in the VH. Taken together, our results suggest that the VH, despite its connection to the paraventricular nucleus of the hypothalamus, may not be a critical site for FKBP51-mediated modulation of the HPA axis. 

Our study has some limitations that should be noted. First, we do not know if female rats would show similar behavioral changes, since a previous study showed that deletion of FKBP51 produces different effects in female mice. Additionally, this study demonstrated that the knockout of FKBP51 in GABAergic versus glutamatergic neurons leads to distinct behavioral outcomes [70]. Although we utilized an AAV5 vector to enable transduction in neurons, the specific neuronal subtype driving the observed effects is still unidentified. Furthermore, the observed impairment in fear recall in our animals made it difficult to assess the effect of FKBP51 knockdown on extinction memory. Lastly, while we suggest that increased GR signaling may be responsible for the memory impairments, we did not directly measure GR signaling in this study. Although our study demonstrates the behavioral effects of reducing FKBP51 expression in the VH, the underlying mechanisms remain unclear. Further investigations into the specific molecular pathways and neural circuits involved could provide deeper insights into how FKBP51 modulates fear learning and memory. 

Taken together, our findings highlight that differential expression of FKBP51 in the VH induces changes in fear learning and memory. We demonstrate that locally reducing FKBP51 in the VH decreased fear memory consolidation. In addition, reducing FKBP51 in the VH did not affect exploratory behavior and locomotion or disrupt the HPA axis response to acute restraint stress. Our results suggest that lower levels of FKBP51 in the VH before a traumatic event may prevent exaggerated responses to fear cues and long-lasting fear memories. 

## 4. Materials and Methods

### 4.1. Animals

Adult male (P60) Sprague Dawley rats (280–310 g) were obtained from the Ponce Health Sciences University colony. They were housed two per cage and maintained under standard conditions with a 12 h light/dark cycle with free access to food and water. The Ponce Health Sciences University Institutional Animal Care and Use Committee (IACUC) approved all the animal work. All research followed the National Institutes of Health’s Guide for the Care and Use of Laboratory Animals

### 4.2. Short Hairpin RNA Construct

The shRNA targeting the rat FKBP5 was driven by a U6 promoter with mCherry as a fluorescent reporter gene. The AAV5 construct contained four different shRNA sequences targeting the FKBP5 mRNA (Clone 1: cgaaccaatgagctta, Clone 2: gcgaggatctatttgaagatt, Clone 3: cgccaacatgttcaagaagtt, Clone 4: cgtgattcagtacgggaagat) for FKBP5 knockdown [18]. As a negative control, a scramble-shRNA (ggaatctcattcgatgcatac) was utilized.

### 4.3. Stereotaxic Surgery

At the time of surgery, adult male rats (P60) weighing around ~280–310 g were placed on stereotaxic apparatus, subcutaneously injected with the analgesic carprofen (5 mg/kg), and anesthetized with isoflurane (3–4%). A volume of 1.0 μL per hemisphere of an AAV5 vector (Charles River Laboratories, Wilmington, MA, USA) expressing either FKBP5-shRNA (AAV5-4-in-1 shRNA to FKBP5 with mCherry) or a scramble-shRNA (AAV5-u6-mCherry-scramble) was injected bilaterally into the VH (−5.6 mm AP, ±4.7 mm ML from bregma, −7.2 mm DV from dura) using a Hamilton syringe at a rate of 0.5 μL/min. After injection, the syringe was left in place for an additional 10 min to allow viral diffusion and avoid backflow. Then, we removed the syringe and sealed the incision site with stitches. After a 30-day recovery period, to ensure adequate viral expression, rats were exposed to behavioral tests. 

### 4.4. Auditory Fear Conditioning and Extinction Training

To test whether reducing VH FKBP5 is sufficient to affect auditory fear conditioning or extinction, adult (P90) male Sprague Dawley rats infused with FKBP5-shRNA or Scramble-shRNA were exposed to classic Pavlovian fear conditioning. All behavioral procedures were conducted in a clear 25.5 × 25.5 × 36 cm Plexiglas chamber (ID#46002, UgoBasile, Gemonio, Italy). The floor in the chamber consisted of stainless-steel bars that provided the shocks (0.50 mA). The chamber was housed in a noise-isolating box, which had a video camera for recording the behavior sessions. In between trials, chambers were cleaned with a 70% ethanol solution. The fear response was measured as the time the rats spent immobile when a tone (30 s) was played. The behavioral procedure consisted of fear conditioning (Day 1), extinction (Day 2), and extinction recall (Day 3). On the first day, they received four tones (30 s, 1000 Hz, 80 dB), one baseline tone and three tone-shock (0.50 mA) pairings in 2 min intervals. The following day, animals were exposed to 14 tones (30 s, 1000 Hz, 80 dB) without shocks, separated by 2 min intervals in the conditioning context. On the third day, rats received two tones (30 s, 1000 Hz, 80 dB) without shocks separated by 2 min in the conditioning context. All videos were recorded and analyzed using the ANY-Maze Software 7.4 (Stoelting Co., Wood Dale, IL, USA). Animals were euthanized 20 min after the extinction recall test. Animals with improper infusion sites were excluded from any behavioral assessment. The ERI was calculated per animal with the following formula: ((Avg Ext 1,2) − (Avg Ext Recall 1,2))/(Max Fear acquired COND) × 100. This index expresses retention test responses as a percentage of acquisition test responses. 

### 4.5. Forced Swim Test (FST)

For each test subject, a cylindrical glass tank (dimensions: 20 cm in diameter × 40 cm tall) was filled with 8.81 L of tap water. The water temperature was adjusted to 27 °C before beginning each trial. The rats were placed individually inside the water tank for ten minutes. Their behavior was recorded by a video camera with the ANY-Maze Sofware 7.4 (Stoelting Co., Wood Dale, IL, USA). After ten minutes, the rats are taken out of the tank, dried with an absorbent bench under pads, and placed under a heating lamp. Videos were manually analyzed, and the experimenter was blind to the treatment of the animal.

For the video analysis, the time each rat spent swimming, diving, struggling, and immobile was quantified. The rat was classified as diving when it submerged and headed towards the bottom of the tank up until it resurfaced. As for struggling, the rat had to flail its front paws above the water, causing it to splash. Immobility was measured when the rat only moved its hind paws slowly or had no movement at all. Any other movements were considered swimming.

### 4.6. Open Field Test (OFT)

Each rat was individually placed on a 45 cm × 45 cm floor with 40 cm high walls made of wood covered with black acrylic. The test was performed under red light conditions. The behavior of the rats was video-tracked with the ANY-Maze Sofware 7.4 (Stoelting Co., Wood Dale, IL, USA) for five minutes. The chamber was cleaned with ethanol between trials. The time the rats spent within the center of the maze, the time they spent moving, and the total distance traveled were measured. 

### 4.7. Elevated Zero Maze Test (EZM)

The maze consisted of a circular track made of black acrylic divided into four sections of equal length. The track measured 11 cm wide and stood 72 cm from the ground. The maze had a diameter of 120 cm. There were two types of alternating sections: open quadrants which consisted of only the track and closed quadrants which had 20 cm high walls on both sides of the track. Each rat was individually placed in the zero maze for five minutes while their behavior was recorded using ANY-Maze Sofware 7.4 (Stoelting Co., Wood Dale, IL, USA). The time they spent in open and closed quadrants and the number of entries into each one were measured. The maze was cleaned with ethanol in between runs.

### 4.8. ELISA Assay of Corticosterone

Rats were restrained continuously for 15 min with DecapiCones, during which blood samples were collected at 0 min and 15 min. Following this, additional samples were taken at two distinct time points: 30 min and 60 min after the initial baseline measurement. For these time points, the animals were briefly restrained (less than 5 min) to obtain blood samples. At the time of the euthanasia, a final blood sample was collected from the heart, 90 min after the initial baseline. Subsequently, samples were centrifuged for serum separation and stored at −80 °C until analyzed. The corticosterone protein was quantified with an enzyme-linked immunosorbent assay (ELISA) kit from Immuno-Biological Laboratories, Inc. (Cat IB79175, Minneapolis, MN, USA). We used a total of 10 μL of serum and followed the protocol according to the manufacturer’s instructions. All the samples were run in replicates.

### 4.9. Immunofluorescence 

Rats that received the viral infusions were euthanized with Euthanasia-III Solution (Pentobarbital Sodium, Phenytoin Sodium, MED-PHARMEX™) after behavioral training and transcardially perfused with saline solution. Brains were fixed with 10% neutral buffered formalin (VWR, 89370-094), embedded in paraffin, and cut into coronal slices at 4 µm in a microtome. We deparaffinized and dehydrated the sections with washes of xylene, alcohol, and PBS. We incubated slices with Citrate-EDTA Buffer at 95 °C for 40 min for antigen retrieval and then with HK112-9KE solution (BioGenex, Fremont, CA, USA) for 15 min for protein blocking in a humidified chamber at 4 °C with primary antibodies against FKBP5 (Abcam, Cambridge, MA, USA, ab126715; 1:500) and mCherry (Neuromics, Edina, MN, USA, MO22192; 1:500). For the secondary antibodies, we used Alexa Fluor^®^ 555 goat anti-rabbit (A-21429, 1:100) and Alexa Fluor^®^ 488 goat anti-mouse (A-11029, 1:100) from Life Technologies, Carlsbad, CA, USA. To prevent fluorescence photobleaching, Prolong Gold antifade reagent (Invitrogen, Waltham, MA, USA, P36934) was added before mounting slices. Images were taken with an Olympus BX60 light microscope (Tokyo, Japan), Nikon digital camera (Nikon DS-Fi1, Melville, NY, USA), and camera control unit (Nikon DS-U2) with the Nikon-NIS element software AR 2.22.25. 

### 4.10. Statistical Analysis

We measured the lack of movement (freezing behavior) during each 30 s tone with the ANY-Maze software. A repeated-measure two-way ANOVA was used for the behavioral analysis using the program GraphPad PRISM 10 by Dogmatics, Boston, MA, USA. Significant ANOVAs were followed by uncorrected Fisher post hoc analysis. A significant difference was considered if *p* < 0.05. Data are presented as mean ± SEM (standard error of the mean). For the OFT and the EZM, we used the ANY-Maze Sofware 7.4 (Stoelting Co., Wood Dale, IL, USA) to measure locomotion and anxiety-like behavior. FST videos were recorded with the ANY-Maze software, and videos were manually analyzed. All the data were grouped and subjected to normality and lognormality tests to ensure their distribution before performing unpaired *t*-tests or Mann–Whitney tests, with *p* < 0.05 as the threshold for significant differences. Data are presented as mean ± SEM. 

## Figures and Tables

**Table 1:** Summary of statistical analysis auditory fear conditioning.

Figure	Measured Condition	Factor	Statistics	Value
Figure 2B	Freezing % during COND	Treatment	Two-way ANOVA	F (1, 17) = 17.21, *p* = 0.007
		Animal		F (17, 23) = 2.69, *p* = 0.004
		Tone		F (19, 323) = 13.79, *p* < 0.001
		Tone x Treatment		F (19, 323) = 2.69, *p* = 0.0002
	Freezing % during COND 3	Treatment	Two-way ANOVA, Uncorrected Fisher’s LSD	*p* = 0.0102
	Freezing % during EXT 1			*p* < 0.0001
	Freezing % during EXT 2			*p* < 0.0001
	Freezing % during EXT 3			*p* = 0.0005
	Freezing % during EXT 4			*p* = 0.0027
Figure 2C	Freezing% during RECALL	Treatment	Mann–Whitney test	*p* = 0.0282
Figure 2D	Extinction Retention Index	Treatment	Mann–Whitney test	*p* = 0.3950

**Table 2:** Summary of statistical analysis for locomotion, exploratory behaviors and forced swim test.

Figure	Measured Condition	Factor	Statistics	Value
Figure 3B–D	OFT: Time in Center	Treatment	Unpaired *t*-test	*p* = 0.1326
	OFT: Distance	Treatment	Unpaired *t*-test	*p* = 0.0654
	OFT: Mean Speed	Treatment	Unpaired *t*-test	*p* = 0.4048
Figure 3E–G	EZM: Time in Open Arms	Treatment	Unpaired *t*-test	*p* = 0.8685
	EZM: Open Arms Entries	Treatment	Unpaired *t*-test	*p* = 0.7898
	EZM: Mean Speed	Treatment	Unpaired *t*-test	*p* = 0.1445
Figure 3H–K	FST: Swimming	Treatment	Unpaired *t*-test	*p* = 0.1309
	FST: Struggling	Treatment	Unpaired *t*-test	*p* = 0.0168
	FST: Diving	Treatment	Unpaired *t*-test	*p* = 0.7489
	FST: Immobility	Treatment	Unpaired *t*-test	*p* = 0.0168

OFT = open field test, EZM = elevated zero maze, FST = forced swim test.

## Data Availability

The raw data supporting the conclusions of this article will be made available by the authors on request.

## Abbreviations

FK506-binding protein 5 (FKBP51); post-traumatic stress disorder (PTSD); Hypothalamic–Pituitary–Adrenal Axis (HPA axis); glucocorticoid receptor (GR); ventral hippocampus (VH); open field test (OFT); elevated zero maze (EZM); forced swim test (FST); short hairpin RNA (shRNA); standard error of the mean (SEM).
